# Role of Interlayer Expansion and Surface Chelation in Ternary NiCuAl Layered Double Hydroxides for Ultrahigh Capacity Dye Adsorption

**DOI:** 10.1002/gch2.70149

**Published:** 2026-08-21

**Authors:** Mrudul Velhal, Raihana Sattari, M. Humaun Kabir, Yu‐Sheng Li, Hong Liang

**Affiliations:** ^1^ Department of Materials Science and Engineering College Station Texas A&M University College Station Texas USA; ^2^ Department of Mechanical Engineering Texas A&M University at Qatar Doha Qatar; ^3^ J. Mike Walker ’66 Department of Mechanical Engineering Texas A&M University College Station Texas USA

**Keywords:** anionic dye adsorption, Eriochrome Black T, interlayer engineering, surface chelation, ternary LDH, water remediation

## Abstract

Developing high‐performance adsorbents for anionic dye removal is essential for sustainable water remediation. However, conventional methods struggle with the persistent, non‐biodegradable nature of synthetic azo dyes. In this study, we report robust hydrothermal synthesis of ternary NiCuAl layered double hydroxide (LDH), exploiting its tunable interlayer chemistry and high surface reactivity for efficient anionic dye capture. Characterization via XRD, XPS, and BET confirmed a mesoporous framework (*D*_pore_ = 3.78 nm) with an expanded interlayer spacing of 8.58 Å, facilitating superior molecular accessibility. Langmuir isotherm fitting indicated the dyes underwent monolayer adsorption with theoretical adsorption capacities (*q*_max_) of 6628 mg g^−1^ for Eriochrome Black T (EBT) and 567 mg g^−1^ for Allura Red AC (AR). Kinetic analysis followed the pseudo‐second‐order model, confirming chemisorption as the primary mechanism. The Weber‐Morris model revealed a multi‐stage uptake governed by film diffusion and pore transport. The remarkable 13‐fold difference in capacity is attributed to the chelation effect of EBT, which acts as a tridentate ligand forming stable complexes with Ni^2+^ and Cu^2+^ cations. DFT analysis indicates that EBT exhibits stronger electrostatic and geometric anisotropy, promoting more directional interactions. Overall, metal coordination and an expanded framework make NiCuAl LDH an exceptional material for high‐capacity wastewater treatment.

## Graphical abstract

An engineered ternary NiCuAl LDH achieves an extremely high *q*_max_ of 6628 mg g^−^^1^ for Eriochrome Black T. The synergy of the 8.58 Å interlayer spacing and robust surface chelation at multi‐metallic centers establishes a new adsorbent‐design framework for the ultra‐high‐performance remediation of bulky anionic pollutants.

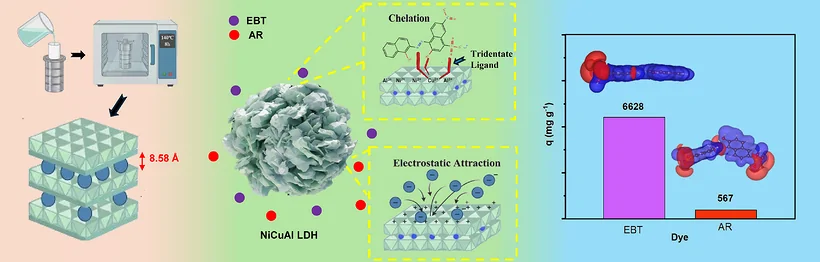

## 1 Introduction

Synthetic dyes are extensively used in textile, food & beverage, paper, and pharmaceutical industries due to their vibrant colors, stability, and low production cost [1]. Their widespread application results in generation of vast amounts of wastewater. Azo dyes represent the largest share of dyes used globally and, by extension, also account for the highest effluent volume [2, 3]. Several of these dyes are non‐biodegradable, toxic, and persistent in aquatic environments, endangering ecosystems and human well‐being. Their presence in wastewater reduces light penetration, disrupts photosynthetic activity, and introduces hazardous chemical residues into the food chain. Ingestion of these harmful chemicals has also shown to have carcinogenic and mutagenic effects in humans [4]. Their complex chemical structures also hinder natural degradation, leading to environmental accumulation. Thus, eliminating wastewater dyes is a critical challenge in environmental remediation.

Among azo dyes, Allura Red AC (AR) and Eriochrome Black T (EBT) were selected as model pollutants for this study on the basis of a deliberate structural contrast. Although the two dyes have comparable molecular weights (496.42 and 461.38 g mol^−1^ respectively) and are anionic in nature, they differ sharply in their capacity for metal coordination. The bulky methoxy group present in AR precludes metal coordination due to steric hindrance [5]. In contrast, EBT forms stable chelation compounds by acting as a tridentate ligand [6]. This pairing thus allows the dyes to act as controls for chelation‐driven adsorption, allowing us to study the impact of chelation to overall adsorption performance.

Efforts in wastewater treatment have been focused on one of several methods, such as adsorption, photocatalysis, filtration, coagulation‐flocculation, ion exchange, and sedimentation [3]. The adsorption approach overcomes several drawbacks of other techniques owing to advantageous characteristics such as low toxicity, affordability, low energy consumption, reusability, and scalability [2]. Studies have utilized adsorbents from a wide array of material classes, such as metal oxides, clays, polymers, and carbon‐based or even hybrid compositions of one or more classes [7, 8, 9]. While these traditional materials show promise, the ever‐evolving requirements for increased theoretical capacities and faster kinetics have shifted focus towards materials that have attributes more than just surface area. Within this context, Layered double hydroxides (LDHs) offer a unique bridge between the chemical robustness of inorganic oxides and the versatility of surface interactions found in carbon‐based materials.

LDHs are a class of 2D materials with brucite‐like structures (Mg(OH)_2_) comprising of alternating sheets of metal cations and interlayer anions. LDHs have a general composition of [M*^2+^*_(1‐_ *_x_*_)_M*^3+^_x_* (OH)_2_]*^x+^* (A*^n^* ^−^) *_x/n_*.mH_2_O, where M^2+^ and M^3+^ are transition metal cations and A^n−^ is the interlayer anionic species such CO_3_^2−^, NO_3_^2−^, or Cl^−^. The partial substitution of M*^2+^* by M*^3+^* generates a net positive charge on the hydroxide layers, which is compensated by the incorporation of charge‐balancing anions and water molecules within the interlayer galleries [10]. This architecture makes LDHs highly versatile, because both the metal–cation composition (M*^2+^*/M*^3+^* identity and ratio) and the interlayer anion can be tuned to tailor properties such as surface chemistry, interlayer spacing, and ion‐exchange behavior. Importantly, LDHs show strong anion‐selectivity trends (e.g. CO_3_^2−^ is more strongly bound to the cation layers than NO_3_^−^), so choosing the interlayer anion can directly affect how readily a dye molecule can be exchanged or intercalated [11]. This structural tunability has enabled LDHs to be widely used in catalysis, electrocatalysis, and photocatalysis by tailoring accessible metal sites and hydroxyl‐rich surfaces [12]. Beyond catalysis, LDHs have been extensively studied for environmental remediation because their positively charged layers and interlayer galleries can immobilize a broad range of inorganic and organic anions from water [10].

A significant volume of recent research focusing on diverse metal combinations in LDHs for dye adsorption applications underscores the importance of the field. For instance, Pan et al. utilized a MgAl LDH to achieve a maximum adsorption capacity of 485.6 mg g^−1^ against the dye Acid Orange 7 [13]. Lei et al. demonstrated the benefits of interlayer anion engineering, wherein the dodecylsulfonate (DS)‐intercalated MgAl LDH exhibited a fourfold increase in maximum adsorption (846.6 mg g^−1^) against Methyl Orange compared to the pristine LDH [14].

Research interest has also increased towards ternary LDH systems to exploit their synergistic benefits. The incorporation of a second M*^2+^* metal cation is a means of introducing a more heterogeneous electronic environment and a higher density of active sites. This structural advantage occurs because a ternary structure offers two distinct coordination environments which broadens the range of anionic species the material can accommodate. In addition, the difference in ionic radii of the cations introduces local lattice strain which can result in expanded interlayer spacing and increased stability. This approach often results in expanded interlayer spacings and improved chemical stability. Recently, Rathee et al. synthesized a ternary NiAlTi LDH with a high dye uptake of 1250 mg g^−1^ against Methyl Orange and 2000 mg g^−1^ against Orange II [15]. Similarly, a graphene/NiMgAl LDH composite developed by Kazeem et al. displayed a high adsorption value of 384.6 mg g^−1^ against Eriochrome Black T [16]. However, to the best of our knowledge, the synergy of a Ni─Cu─Al framework optimized by nitrate‐mediated interlayer modification remains unexplored for enhanced capture of bulky azo pollutants.

In this study, we report the synthesis of a highly efficient ternary NiCuAl LDH, synthesized via a facile hydrothermal treatment and specifically engineered for the remediation of anionic dye‐tainted wastewater. Utilizing AR and EBT, we systematically evaluated how ternary metal coordination and expanded interlayer distance overcome the sluggish adsorption characteristics associated with anionic dyes. The morphological and structural integrity, as well as the chemical composition, were comprehensively investigated using SEM, XRD, XPS, and BET analysis. The influences of various parameters such as solution pH, adsorbent dosage, starting dye concentration, and contact time were explored, and the equilibrium and kinetic behavior were studied using theoretical models. To provide a molecular‐level basis for the contrasting adsorption performance, DFT calculations were conducted to quantify the electrostatic and geometric anisotropies of the azo dyes. Finally, the possible adsorption mechanisms are discussed in detail.

## 2 Materials and Methods

### 2.1 Materials

Nickel(II) nitrate hexahydrate (Ni(NO_3_)_2_·6H_2_O), copper(II) nitrate trihydrate (Cu(NO_3_)_2_·3H_2_O), and aluminum nitrate nonahydrate (Al(NO_3_)_3_·9H_2_O) were used as metal precursors. Urea (CO(NH_2_)_2_) served as the precipitating and reducing agent. The dyes Eriochrome Black T (EBT) and Allura Red AC (AR) were selected as representative anionic dyes due to their industrial and environmental relevance. Their structures are shown in Figure S1. All materials were sourced from Sigma‐Aldrich (USA). All chemicals were of analytical grade and were used without further purification. Deionized water was used in all solution preparations.

### 2.2 Hydrothermal Synthesis of NiCuAl LDH

**FIGURE 1:**
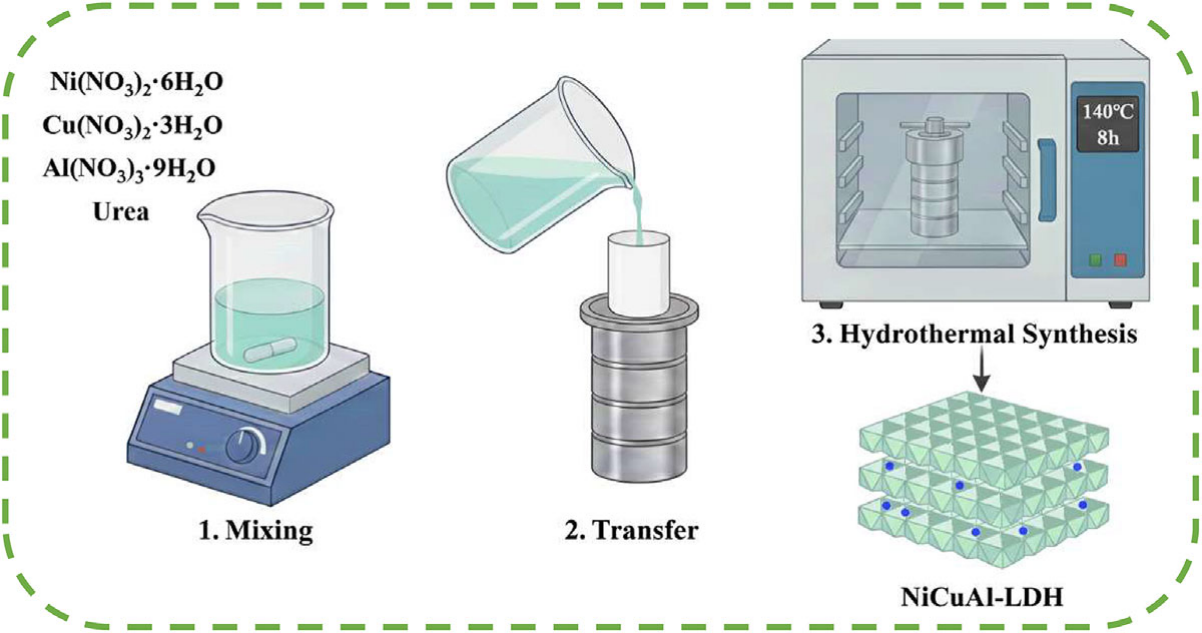
Hydrothermal synthesis procedure of NiCuAl LDH.

LDH was synthesized using a facile hydrothermal method. Ni(NO_3_)_2_·6H_2_O, Cu(NO_3_)_2_·3H_2_O, and Al(NO_3_)_3_·9H_2_O were dissolved in deionized water in a molar ratio of 3:2:2 to form a homogeneous precursor solution. To this solution, 6 mmol of urea was added to facilitate slow hydrolysis, ensuring controlled precipitation and uniform crystal growth [17]. The mixture was magnetically stirred until fully dissolved. After the solution was added to a Teflon‐lined stainless‐steel autoclave, it was sealed and maintained at 140°C for 8 h. After cooling to room temperature, the product was filtered and washed thrice with deionized water and ethanol. The precipitate was dried at 60°C overnight and stored in airtight containers until use. Figure 1 shows the synthesis procedure of the NiCuAl LDH.

### 2.3 Material Characterization

The material properties of the synthesized NiCuAl LDH were evaluated by a variety of characterization techniques. The surface morphology of the LDH was observed by scanning electron microscopy (SEM) on a JEOL JSM‐7500F FE‐SEM instrument with an accelerating voltage of 2 kV. A Bruker D8 Advance X‐ray diffractometer (XRD) was used to characterize the crystal structure and crystallinity. The specific surface area of the LDH was measured using the Brunauer‐Emmett‐Teller (BET) method on an Anton Paar Nova 600. The chemical and atomic composition was studied using an Omicron XPS/UPS system.

In aqueous systems, the adsorption capacity can be tuned by manipulating the surface charge on the adsorbent. A positive net surface charge makes the dye more amenable to anionic dyes, while a negative charge is crucial for adsorbing cationic dyes. The point of zero charge (pH_PZC_) is the pH at which the net surface charge is zero [18]. The pH_PZC_ was measured by employing a salt addition method. The initial pH of a 0.1 m KNO_3_ solution was adjusted between 2–10 in separate flasks and 25 mg of LDH was added. After stirring for 24 h, the change in the pH, ΔpH = (pH_f_‐pH_i_), was calculated. The pH_PZC_ is the point at which ΔpH = 0.

### 2.4 Batch Adsorption Experiments

The batch adsorption tests utilized to assess the adsorption characteristics of the NiCuAl LDH included studying the effects of variations in solution pH, adsorbent dosage, initial dye concentration, and contact time. Equilibrium adsorption isotherm and kinetic models were used to reveal the mechanisms of adsorbent–adsorbate interactions. All tests were carried out at 25 ± 2°C in conical flasks and were done in triplicate to ensure replicability. The importance of strong agitation in the early stages of adsorption to improve the process kinetics was shown by Bayomie et al. [19]. As a result, the mixture was agitated for a fixed period of 120 min followed by magnetic stirring at 200 rpm for the remainder of the tests. The EBT test volume was set to 50 mL while the AR volume was 25 mL per flask. The process was monitored by means of UV–Vis spectroscopy.

Initially, the optimum solution pH was identified by varying the pH from 3 to 11 for an adsorbent dosage of 10 mg and dye concentrations of 200 mg L^−1^ (AR) and 1500 mg L^−1^ (EBT). Upon identification of the optimum test pH, the dosage dependency of the adsorption process was verified by testing the two dyes against LDH dosages between 5 and 25 mg. Then, the adsorbent–adsorbate interactions were examined by studying the adsorption isotherms for both AR‐LDH and EBT‐LDH systems. In addition, an initial dye concentration of 200 mg L^−1^ (AR) and 1500 mg L^−1^ (EBT) was treated by 10 mg of LDH for different durations (5–1440 min) to evaluate the adsorption kinetics. The thermodynamic characteristics of the adsorption process were tested by varying the temperature between 25°C and 55°C. Finally, the reusability of the LDH against both dyes was tested over 3 cycles. The spend LDH was recovered by centrifugation and then stirred overnight in a 1:1 water–ethanol solution containing 0.1 m NaOH for regeneration.

### 2.5 Density Functional Theory (DFT) Calculations

Density functional theory (DFT) calculations were carried out to elucidate the electrostatic potential distribution and geometric anisotropy of representative azo dyes, namely Allura Red AC (AR) and Eriochrome Black T (EBT). All calculations were performed using the Quantum ESPRESSO package. All calculations were performed using the Quantum ESPRESSO package.

The electronic exchange–correlation interactions were described using the Perdew–Burke–Ernzerhof (PBE) generalized gradient approximation. Core–valence interactions were treated with norm‐conserving RRKJ pseudopotentials for all constituent elements (C, H, N, O, and S). A plane‐wave kinetic energy cutoff of 40 Ry was employed for the wavefunctions and 350 Ry for the charge density, ensuring convergence of the total energy and electrostatic properties. Isolated dye molecules were modeled using a rectangular simulation cell, providing a minimum vacuum spacing of ≥15 Å in all directions to eliminate spurious interactions between periodic images. Brillouin zone sampling was restricted to the Γ‐point, appropriate for molecular systems. Long‐range dispersion interactions were accounted for using the Grimme D3 van der Waals correction.

Structural optimization was performed using the Broyden–Fletcher–Goldfarb–Shanno (BFGS) algorithm until the forces on all atoms were minimized, followed by self‐consistent field (SCF) calculations on the optimized geometries. A fixed occupation scheme was used, and electronic convergence was achieved with a threshold of 10^−8^ Ry. Electrostatic potential (ESP) maps were generated from the converged charge density using the post‐processing module (pp.x) of Quantum ESPRESSO.

## 3 Results and Discussion

### 3.1 Characterization of NiCuAl LDH

**FIGURE 2:**
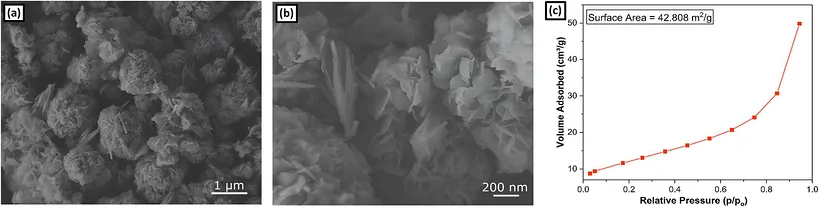
(a, b) SEM images of NiCuAl LDH and (c) N_2_ adsorption plot for BET surface area of NiCuAl LDH.

The morphological analysis of the as‐synthesized LDH was performed using scanning electron microscopy (SEM). Figure 2a,b presents the low and high‐magnification images of the LDH surface. It is observed that the LDH comprises tiny platelet‐like structures that aggregate to form flower‐like structures. Figure 2a illustrates the high porosity structure of the LDH, where the individual aggregates appear as compact spherical flower‐like structures that stack onto each other to form a rough surface. As seen in Figure 2b, the individual platelets appear to have distinct sizes and undergo interlocking to form the flower‐like aggregates. From Figure S2, the average thickness of the platelets was found to be ≈52 nm. The high porosity and surface area of the LDH is derived from the arrangement of the lamellae, which lead to large internal voids.

The BET surface area, average pore size, and pore volume were calculated. As shown in Figure 2c, the N_2_ adsorption plot exhibits a Type IV isotherm according to the IUPAC classification, revealing a mesoporous structure [20]. The significant capillary uptake at high relative pressures (*p*/*p*_0_ > 0.8) suggests capillary condensation within the mesopores, which is a common feature in LDHs [21, 22]. The LDH possessed a BET specific surface area of 42.81 m^2^ g^−1^. Using the t‐plot method, the external surface area was calculated to be 38.33 m^2^ g^−1^, which implies that a majority of the porosity is derived from the surface level and accessible mesopores rather than internal micropores. The pore size distribution obtained using the NL‐DFT method (Figure S3) showed a prominent peak at 3.78 nm, confirming the mesoporous nature of the LDH [23]. The total pore volume was determined to be 0.0685 cm^3^ g^−1^. The high porosity and accessible active sites on the surface facilitate better adsorption properties [24].

**FIGURE 3:**
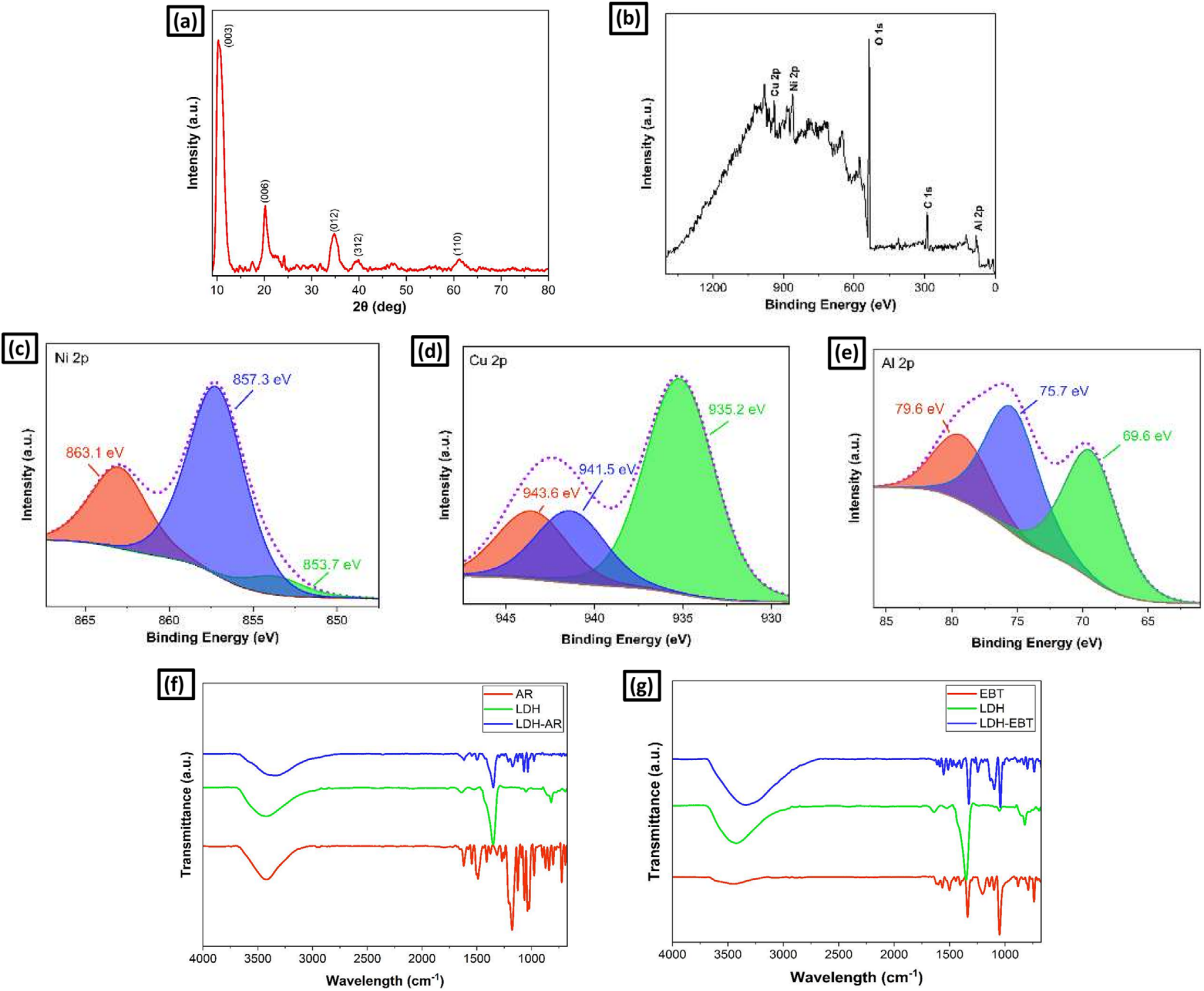
(a) XRD pattern, and (b) XPS survey spectrum of NiCuAl LDH; (c) Ni 2p, (d) Cu 2p, and (e) Al 2p high resolution spectrum; FTIR spectra before and after batch adsorption experiments of (f) AR‐LDH and (g) EBT‐LDH systems.

The crystalline phase and structural parameters of the hydrothermally synthesized NiCuAl LDH were studied using XRD. As illustrated in Figure 3a, the XRD pattern exhibits characteristic reflections of a well‐defined hydrotalcite‐like structure. Sharp peaks at 2*θ* values of 10.3^°^, 20.3^°,^ and 34.7^°^, which are ascribed to the (003), (006), and (012) basal planes, respectively [14, 25]. The symmetric (110) reflection at 60.1° further confirms the formation of a layered framework with rhombohedral symmetry [26]. Additionally, minor reflections are observed at 15.1^°^ and 17.5^°^, which suggest the presence of minor localized disorder and secondary hydroxide domains. This distortion can be caused by the Jahn‐Teller effect due to the presence of Cu^2+^ in the matrix [27, 28].

To quantify the structural dimensions, we utilized Bragg's Law (*nλ* = 2*d*sin *θ*) to determine the interlayer spacing (*d*‐spacing). For the (003) plane at 10.3^0^, the *d*‐spacing was calculated to be 8.58 Å. Notably, this value is significantly higher than the *d*‐spacing of typical carbonate‐intercalated LDHs, which generally exhibit a (003) reflection at approximately 11.6^0^ (*d* ≈ 7.6 Å) [29, 30]. This interlayer expansion is attributable to the co‐intercalation of nitrate (NO_3_^−^) and carbonate (CO_3_^2−^) anions. The presence of a low‐intensity (006) peak at 24.1^°^, which would normally correspond to a pure CO_3_^2−^‐intercalated interlayer supports this conclusion. The larger hydration radius of the nitrate ions (0.316 nm), relative to pure carbonate (0.265 nm), introduce a wider interlayer spacing, thus enhancing the accessibility of internal active sites to bulky anionic dye molecules like EBT [31, 32].

The unit cell was also characterized by calculating the lattice parameters “a” and “c” (*a* = 2*d*_110_; *c* = 3*d*_003_). The “a” parameter represents the average cation–cation distance in the unit cell and was calculated to be 3.08 Å. The “c” parameter is related to the interlayer thickness and was calculated to be 25.7 Å. Finally, the average crystallite size was calculated to be 17.3 nm using the Debye Scherrer equation, which is within the reported limits for LDH crystallite sizes [20]. The robust crystallite size, coupled with the expanded interlayer distance, signifies the material is well‐suited to accommodate dye molecules during the adsorption process.

X‐ray photoelectron spectroscopy (XPS) was used to investigate the elemental composition and valence states of the synthesized LDH. The CasaXPS software was used to analyze the raw data [33]. Figure 3b–e describes the survey spectra of the LDH, and the high‐resolution spectra of Ni 2p, Cu 2p, and Al 2p, respectively. In Figure 3b, the survey spectrum confirms distinct photoemission lines for Ni, Cu, Al, C, and O. The O 1s spectrum signal corresponds to the metal‐hydroxide (M─OH) bonds of the LDH layers. Figure 3c shows the high‐resolution spectrum for Ni 2p. The characteristic peaks shown at 853.7 and 857.3 eV both originate from Ni 2p_3/2_, while a shake‐up peak is observed at 863.1 eV [34, 35]. These binding energies are consistent with Ni^2+^ ions, which correspond to the Ni(OH)_2_ present in the LDH structure. In Figure 3d, the primary characteristic peak of Cu 2p was observed at 935.2 eV, thus confirming the Cu 2p_3/2_ state and the Cu^2+^ species in the LDH matrix. Further, two strong satellite peaks were also observed at 941.5 and 943.6 eV [30, 36]. Finally, Figure 3e presents the high‐resolution spectrum for Al 2p. Upon deconvolution, three distinct peaks at 75.7, 69.6, and 79.6 eV were observed. The 75.7 eV peak is consistent with Al^3+^ ions in octahedral coordination [30]. However, the 69.6 and 79.6 eV peaks were not related to Al 2p and were instead the result of spectral overlap from Ni 3p and Cu 3p, respectively [37, 38].

The successful adsorption of the dyes in the AR‐LDH and EBT‐LDH systems is confirmed by the FTIR spectra presented in Figure 3f,g respectively. The pristine LDH spectrum exhibits a sharp peak at 1350 cm^−1^, which corresponds to a combination of the CO_3_^2−^ and NO_3_^−^ interlayer anions [39, 40]. The broad peak centered at 3421 cm^−1^ corresponds to the OH^−^ groups in the layers. The peak at 822 cm^−1^ can be attributed to the M─O metal─oxide bond in the LDH structure [29]. In Figure 3f, the FTIR spectrum of Allura Red displays the traditional peaks associated with the dye, namely the 1178 cm^−1^ and 1038 cm^−1^ peaks associated with SO_3_^−^ asymmetric and symmetric stretching and ─N═N─ azo peak at 1488 cm^−1^ [41]. Figure 3g shows the FTIR spectrum of the EBT‐LDH system. In the unreacted dye, the peaks at 1337 cm^−1^ and 1200 cm^−1^ represent the C─N and C─O stretching vibrations respectively, while the strong 1050 cm^−1^ peak is again representative of the SO_3_^−^ stretching. Other notable peaks include the N─O peak at 1500 cm^−1^ and the out‐of‐plane C─H bending at 791 and 734 cm^−1^ [42].

In both dye systems, the post‐adsorption spectra show the presence of functional groups of their respective dyes, which confirms successful adsorption. In the AR‐LDH system, the azo peak at 1488 cm^−1^ significantly decreases in intensity, suggesting that the azo group is a primary participant in the adsorption process, likely through electrostatic interactions with the positively charged metal hydroxide sites [43]. Similarly, the attenuation of the SO_3_^−^ stretching peaks at 1178 and 1038 cm^−1^ is evidence of the formation of coordination bonds with LDH layers. This is supported by the disappearance of the M─O peak at 822 cm^−1^ seen in the LDH spectrum [44, 45]. The shift of the LDH CO_3_^2−^ and NO_3_^−^ peak at 1350 cm^−1^ to 1345 cm^−1^ confirms the perturbation of the interlayer anions, possibly due to their displacement by the adsorption dye molecules. Similar trends are observed in the EBT‐LDH system, where the SO_3_^−^ peak at 1050 cm^−1^ is diminished due to metal coordination and the 1337 cm^−1^ peak shifts to 1326 cm^−1^. Uniquely, a C─O peak at ≈1100 cm^−1^ gains in intensity post‐adsorption for the EBT‐LDH, which is possibly a result of the strong surface coordination bonds between EBT hydroxide groups and LDH metal sites [46].

### 3.2 Batch Absorption Studies

The adsorption process can be substantially affected by the solution pH due to the charged moieties present on both the dye and the adsorbent molecules. To this end, the effect of varying solution pH was studied for solutions of both AR and EBT dyes, where the pH varied between 3 and 11. The dosage was kept constant at 10 mg, while the dye concentration was maintained at 200 and 1500 mg L^−1^ respectively for the AR and EBT dyes. For both dye systems, the maximum adsorption was observed at pH 3 (image not shown), with the degree of dye removal gradually falling off with increasing pH. This can be explained by examining the relation between the solution pH and the pH_PZC_ of the NiCuAl LDH. From Figure S4, the pH_PZC_ was found to be 6.2. At pH values below the pH_PZC_, the surface is positively charged. The protonation of the LDH surface leads to a high density of positive charges, leading to enhanced electrostatic attraction with the negatively charged dye anions. Conversely, increasing the solution alkalinity above the pH_PZC_ saturates the LDH surfaces with OH^−^ ions [47]. This reduces the adsorption amount due to electrostatic repulsion with the anionic dye molecules. As a result, pH 3 was chosen as the ideal pH for the rest of the batch adsorption experiments.

The relation between adsorption amount and dosage is crucial in determining the efficiency and economic viability of the adsorbent. In this test, the dye concentrations were kept constant at 200 mg L^−1^ for the AR and 1500 mg L^−1^ for the EBT, while the dosage was varied between 5 and 25 mg. The amount of adsorption (q) and the % removal (R(%)) were calculated as

(1) $$q=\frac{V\left(C_{o}-\;C_{e}\right)}{m}$$

(2) $$R\left(\%\right)=\frac{C_{o}-\;C_{e}}{C_{o}}\;\times 100$$

where *C*_0_ (mg L^−1^) and *C*_e_ (mg L^−1^) signify the initial and the equilibrium dye concentration, and V and m are the dye volume and mass of the adsorbent, respectively.

**FIGURE 4:**
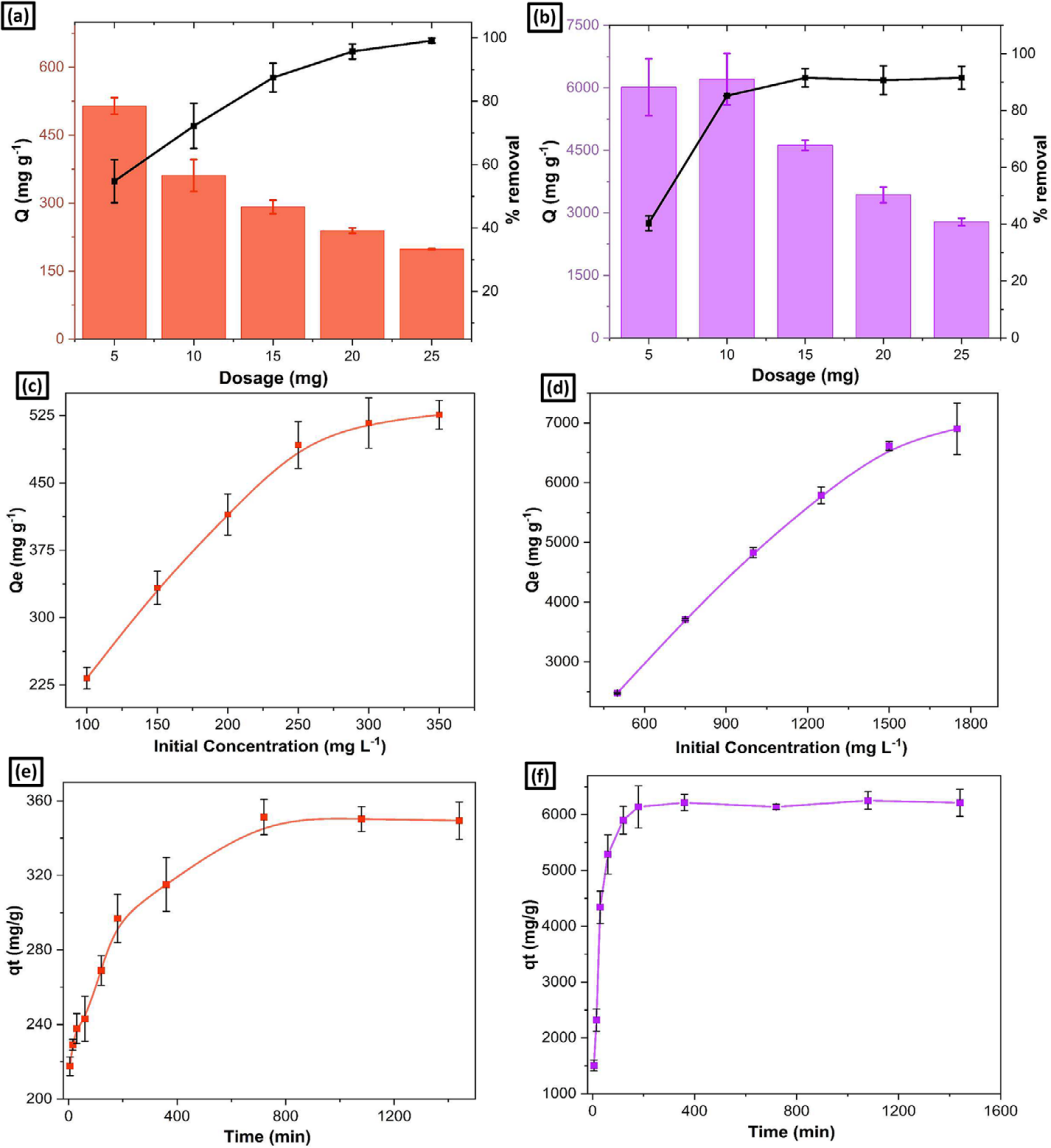
Variation of *q* and % removal for (a) AR dye (initial dye conc.: 200 mg L^−1^, dosage range: 5–25 mg, pH: 3), and (b) EBT dye (initial dye conc.: 1500 mg L^−1^, dosage range: 5–25 mg, pH: 3); dye concentration dependency profile for (c) AR dye (dosage: 10 mg, pH: 3, dye conc.: 100–350 mg L^−1^) and (d) EBT dye (dosage: 10 mg, pH: 3, dye conc.: 500–1750 mg L^−1^); time‐dependent adsorption profile for (e) AR dye (dosage: 10 mg, dye conc.: 200 mg L^−1^; duration: 5–1440 min) and (f) EBT dye (dosage: 10 mg, dye conc.: 1500 mg L^−1^; duration: 5–1440 min).

The dye dependence results illustrated in Figure 4a,b demonstrate a clear increase in % removal with increasing dosage for both AR and EBT systems. The LDH showed a maximum removal efficiency of 99% for the AR and 92% for the EBT at a dosage of 25 mg. The % removal increases rapidly with an initial increase in dosage, which is attributed to a greater availability of active sites for adsorption. The eventual plateau is a result of saturation of the active sites by dye molecules and suggests a monolayer adsorption profile for the LDH [48]. For the AR dye (Figure 4a), the adsorption amount *q* varied between 198.29 ± 1.46 and 568 ± 68.2 mg g^−1^. The highest value of *q* was observed at the lowest dosage of 5 mg and the value fell as the dosages increased. The corresponding adsorption range for the EBT system (Figure 4b) was 2782 ± 89.6 to 6209 ± 619.4 mg g^−1^. At lower dosages, the limited number of active sites ensures complete coverage by the adsorbed molecules. As the dosage increases, the surfeit of available sites leads to vacant sites and an overall unsaturated surface, which causes a dilution of the adsorption amount. This observation is mathematically supported by Equation (1), where *q* and the mass *m* bear an inverse relation. Additionally, a potential screening effect caused by particle aggregation at higher dosages may reduce the effective accessible active sites, thus suppressing the *q* value [49].

Another important consideration is the effect of initial dye concentration on the adsorption parameters. In this test, the LDH dosage was maintained at 10 mg throughout and the initial dye concentrations were varied from 100–350 mg L^−1^ (for AR) and 500–1750 mg L^−1^ (for EBT). Figure 4c,d depict the adsorption trends for AR‐LDH and EBT‐LDH systems. The maximum adsorption amount was 525 ± 16 mg g^−1^ and 6900 ± 430 mg g^−1^ respectively. Both plots show a rapid increase in *q* at lower concentrations with an eventual plateau formation at higher concentrations. This particular trend is the result of two competing phenomena. The rapid increase in the dye uptake in the initial stages is ascribed to the mass concentration gradient between the dye and adsorbent molecules, which acts as a driving force for adsorption. However, the subsequent plateauing is the result of saturation of the limited number of adsorption sites [19, 50].

Further studies were carried out to evaluate the influence of contact time on adsorption capacity. 10 mg of LDH were added to 200 mg L (AR) and 1500 mg L (EBT) and agitated for 5–1440 min. Figure 4e,f illustrates the variation of the adsorption amount at time t (*q_t_*) over time. The kinetic profiles exhibit biphasic behavior in both systems which produce a rapid initial increase in adsorption followed by plateauing. The AR‐LDH system achieved equilibrium within 800 min, while the EBT‐LDH system was significantly quicker at 180 min. This initial burst can be put down to the instantaneous availability of active sites.

A comprehensive equilibrium isotherm study was performed to elucidate the interaction mechanisms and describe the adsorption pathways and capacities at equilibrium. The equilibrium data provide critical insights into the surface properties and affinity of the LDH towards the AR and EBT dyes. Two widely utilized models, the Langmuir and Freundlich isotherms, were employed to describe the adsorption process.

The Langmuir model describes a monolayer adsorption profile on a homogeneous surface where all active sites are energetically identical and possess equal affinity for the solute. This model assumes the absence of any intra‐adsorbate interactions. The linear form of the Langmuir equation is as follows:

(3) $$\frac{C_{e}}{q_{e}}=\frac{1}{K_{L}q_{m}}+\frac{C_{e}}{q_{m}}$$

where *q*_e_ (mg g^−1^) is the amount of dye adsorbed at equilibrium, *q*_m_ (mg g^−1^) is the theoretical maximum monolayer adsorption capacity, and *K*_L_ (L mg^−1^) is the Langmuir constant. *K*_L_ serves as a direct measure of the adsorbent‐adsorbate affinity, with a higher *K*_L_ value signifying greater binding energy. The favorability of the adsorption process was evaluated using the dimensionless separation factor (*R*_L_), derived from the Langmuir constant as follows:

(4) $$R_{L}=\frac{1}{1+K_{L}C_{0}}\;$$

In contrast, the Freundlich isotherm is an empirical model that assumes multilayer and heterogeneous adsorption on active sites of varying affinities. It is expressed in its linear form as:

(5) $$\log\;\;q_{e}=\log\;K_{F}+\left(1/n\right)\;\log\;C_{e}$$

where *K*_F_ ((mg g^−1^) (L mg^−1^)^1/^ *^n^* represents the Freundlich adsorption constant and adsorption intensity *n* denotes the favorability of adsorption when 0.1 < *n* < 1.

**FIGURE 5:**
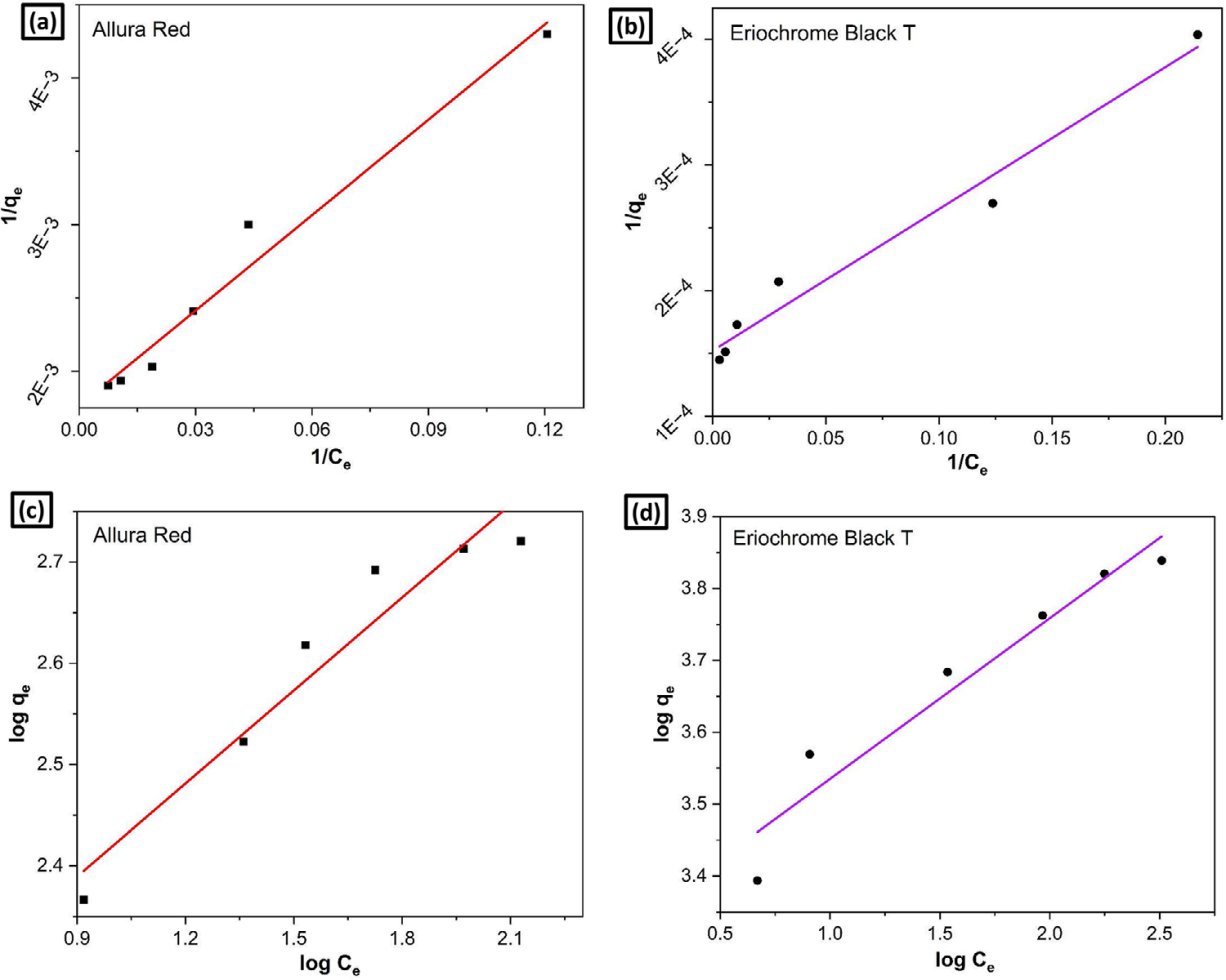
Langmuir plots of the isotherms for (a) AR dye and (b) EBT dye; Freundlich plots of the isotherms for (c) AR dye and (d) EBT dye.

The equilibrium data for both dye systems showed a superior fit to the Langmuir model (*R* ^2^ = 0.966 for AR and 0.957 for EBT) as summarized in Figure 5 and Table 1. In contrast, the R^2^ values for the Freundlich isotherm were relatively lower, at 0.931 and 0.917 for the AR and EBT systems respectively. This high correlation with the Langmuir isotherm suggests that the NiCuAl LDH is predominantly characterized by a monolayer adsorption profile with a finite number of equally energetic active sites. This observation logically follows from the structure of the LDH where the positive charges are uniformly distributed over the layer surface. Moreover, this observation is also consistent with the plateau formation observed at the latter stages of the concentration and time dependency studies, where the adsorption process is effectively ended once all active sites reach saturation.

**TABLE 1:** Equilibrium isotherm fitting parameters for AR‐LDH and EBT‐LDH systems.

Isotherm	Parameter	Value	
		AR	EBT
Langmuir	*q*_m_ (mg g^−1^)	567.01	6628.66
	*K*_L_ (L mg^−1^)	0.081	0.132
	*R*_L_	0.109	0.070
	*R* ^2^	0.966	0.966
Freundlich	1/*n*	0.306	0.223
	*K*_F_ (L mg^−1^)	129.92	2049
	*R* ^2^	0.931	0.917

The *q*_max_ value derived from the Langmuir isotherm was calculated to be 567.01 mg g^−1^ for the AR‐LDH system. This value aligns closely with the experimentally observed *q*_e_ value of ≈525 mg g^−1^. The EBT‐LDH system exhibited a remarkable *q*_max_ of 6628.66 mg g^−1^, which is close to the experimental *q_e_* value of 6900 ± 430 mg g^−1^. While 6628 mg g^−1^ exceeds the typical range reported for LDH‐based dye adsorbents, comparably high gravimetric capacities have been reported for other layered, ion‐exchange‐active nanomaterials. Yuan et al. reported a Langmuir‐fitted maximum capacity of 3937 mg g^−^^1^ for methylene blue on 2D titanate nanosheets, attributing the high uptake to accessible interlayer ion‐exchange sites within a structurally stable layered framework rather than multilayer surface stacking [51]. The present system similarly derives its high capacity from the substantial number of accessible coordination and ion exchange sites. The vast improvement in the adsorption of EBT can be attributed to several factors, including molecular size, charge distributions, and potential for stronger π–π interactions between EBT and LDH, which shall be discussed in detail in the following sections.

The separation factor *R*_L_ also elucidates the differences in affinities for the individual dyes. The calculated *R*_L_ values for AR (*R*_L_ = 0.109) and EBT (*R*_L_ = 0.070) confirm the high favorability for adsorption for both dyes. Notably, the EBT dye has a significantly lower *R*_L_ value, underscoring its higher affinity for the NiCuAl LDH molecules.

The experimental data was evaluated with three distinct adsorption models viz. the pseudo first order (PFO), the pseudo second order (PSO), and the Weber Morris intraparticle diffusion (IPD) to describe the rate‐controlling steps and time dependent behavior of the adsorption. The PFO model, typically associated with physical adsorption, is expressed in its linear form as:

(6) $$\ln\left(q_{e}-\;q_{t}\right)=\ln\;q_{e}-\;K_{1}t\;$$

where *q_t_* (mg g^−1^) is the amount of adsorption at time *t* and *K*_1_ (min^−1^) is the PFO rate constant. In this model, the diffusion of the adsorbate molecules to the adsorbent surface is the rate‐limiting step. Conversely, the PSO model assumes the process to be governed by chemisorption, where the availability of active sites for chemical bond formation is the rate‐limiting step. It is denoted in its linear form as:

(7) $$\frac{t}{q_{t}}=\frac{1}{K_{2}q_{e}^{2}}+\frac{t}{q_{e}}$$

where *K*_2_ (g mg^−1^ min^−1^) is the PSO rate constant which describes the rate at which the system approaches surface saturation.

The Weber Morris IPD model is an integral step in studying adsorption mechanisms. It is given by Equation (8) and details the number of stages in the overall process.

(8) $$q_{t}=\;K_{id}t^{0.5}\;+C$$

where *k*_id_ (mg g^−1^ min^−1/2^) is the diffusion rate constant and *C* represents the boundary layer effect.

**FIGURE 6:**
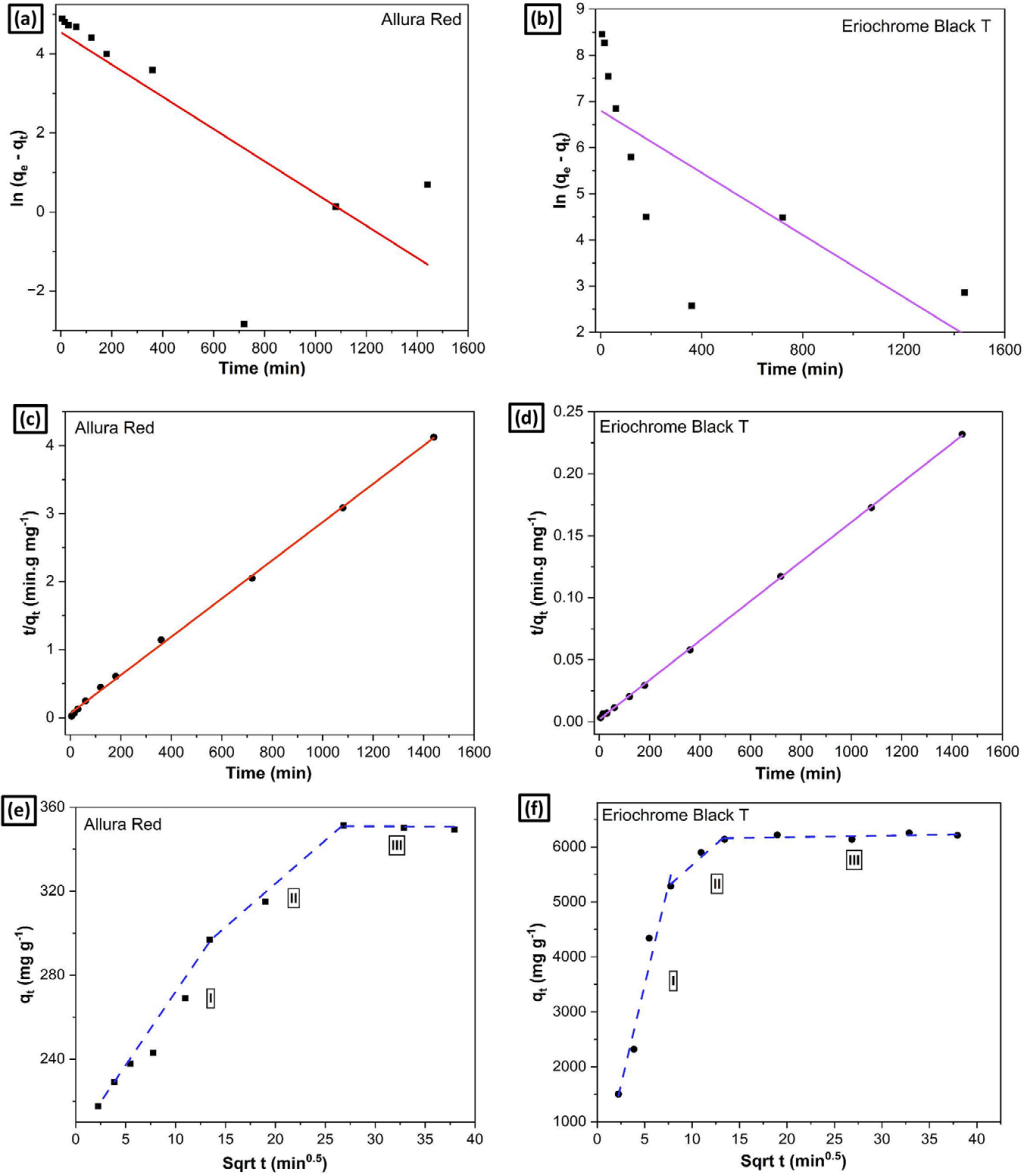
Pseudo first order kinetic plots for (a) AR dye and (b) EBT dye; Pseudo second order kinetic plots for (c) AR dye and (d) EBT dye; Weber‐Morris Intraparticle Diffusion plots for (e) AR dye and (f) EBT dye.

As seen in Figure 6a–d and Table 2, the PSO model yields an extremely high correlation for both dye systems (*R* ^2^ > 0.99), which is markedly better than the PFO model (*R* ^2^ < 0.6). This confirms that chemical bond formation is the primary mechanism of adsorption, presumably due to the strong electrostatic attraction between the brucite‐like layers of the LDH and the dye anions. A noteworthy observation is the variation between the calculated and experimental *q_e_* values. While the *q_e_* value for the EBT system (6290.33 mg g^−1^) is close to the experimentally observed number of 6900 ± 430 mg g^−1^, the AR system yields a significantly lower *q_e_* value of 355.51 mg g^−1^ compared to its experimental value of 525 ± 16 mg g^−1^. Despite the high R^2^ value, this deviation coupled with the high equilibrium time of 800 min (as noted in a previous section) suggests the AR molecules may undergo a secondary, slower uptake phase not fully captured by the PSO model.

**TABLE 2:** PFO and PSO kinetic fitting parameters for AR‐LDH and EBT‐LDH systems.

Model	Parameter	Value	
		AR	EBT
PFO	*q_e_* (mg g^−1^)	94.34	899.56
	*K*_1_ (min^−1^)	.0041	.0034
	*R* ^2^	0.562	0.451
PSO	*q_e_* (mg g^−1^)	355.51	6290.33
	*K*_2_ (g mg^−1^ min^−1^)	1.25 × 10^−4^	1.28 × 10^−5^
	*R* ^2^	0.999	0.999

Further insights into the mass transfer mechanisms were obtained from the IPD plots (Figure 6e,f). In both AR‐LDH and EBT‐LDH systems, the IPD plots display three linear segments, indicating the adsorption process proceeds via three distinct steps with different rates. The first, highly‐sloped region corresponds to the rapid transport of dye molecules from the bulk solution to the external LDH surface. It is important to note the distinction in the first linear regions of both plots; while the AR‐LDH system shows a shorter region with a lower slope, the EBT curve has a much larger and steeper linear region. This suggests that most of the adsorption process in the case of the AR concludes on the surface itself, rather than further penetrating into the interior. The second, slower step in the process is the intraparticle diffusion, which is the rate‐limiting step in both cases. In the case of AR, the longer equilibrium time coupled with slow of intraparticle diffusion suggests that the process continues well beyond this window. As the PSO fitting primarily relies on the initial stages, this would explain the large deviation between the *q_e_* and the experimentally observed *q*. Finally, the third region is nearly flat and depicts the saturation of the LDH, indicating the system is approaching equilibrium with minimal subsequent uptake.

**FIGURE 7:**
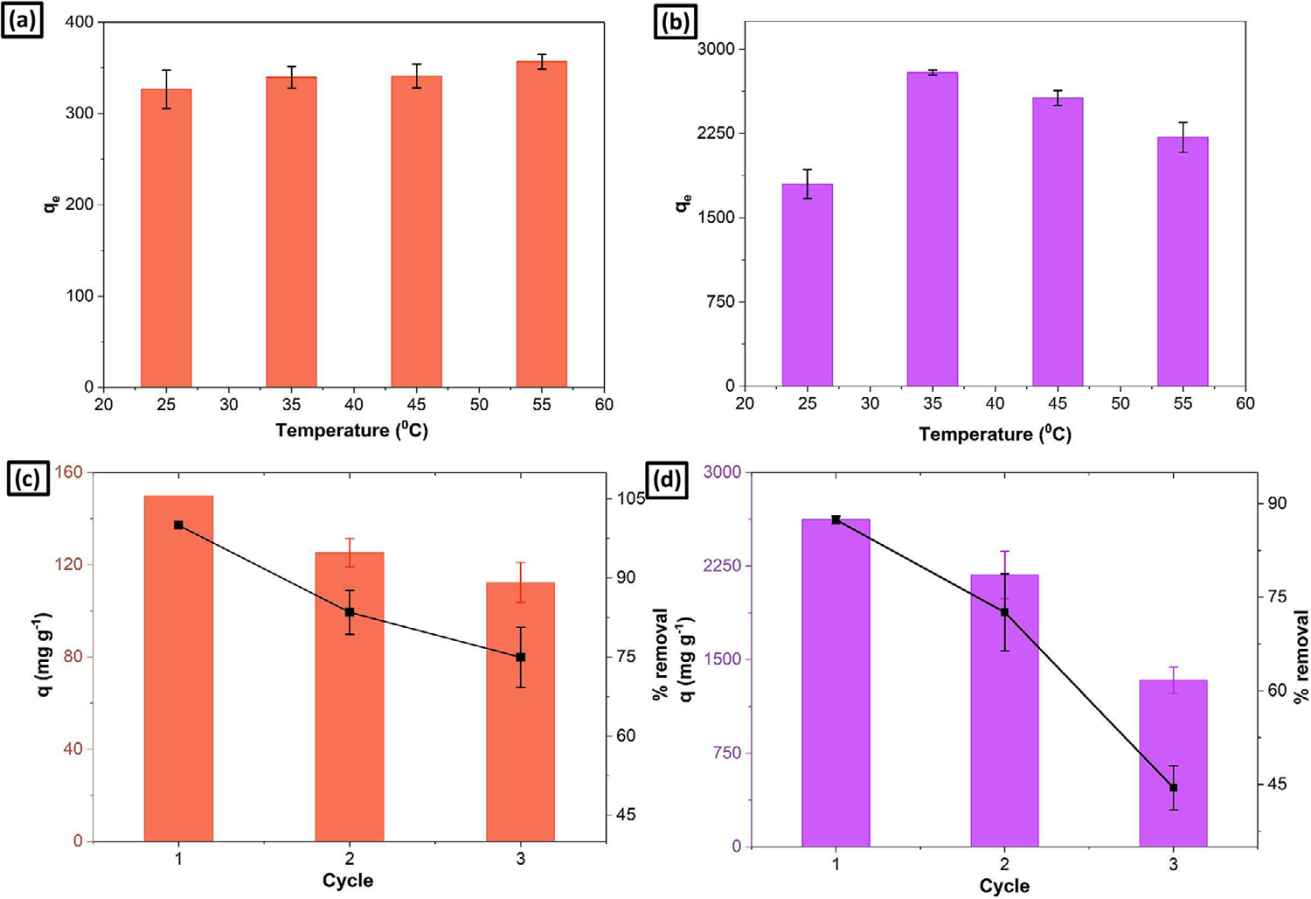
Temperature‐dependance plots for (a) AR (Initial dye conc.: 150 mg L^−1^, dosage: 10 mg, pH: 3, temperature range: 25–55°C) and (b) EBT (initial dye conc.: 1500 mg L^−1^, dosage: 25 mg, pH: 3, temperature range: 25–55°C); reusability plots for (c) AR and (d) EBT over 3 cycles.

The thermodynamic testing was carried out at temperatures between 25°C and 55°C at dye concentrations of 150 mg L^−1^ for AR and 1500 mg L^−1^ for EBT. The variation in *q* across the tested temperatures is shown in Figure 7a,b respectively for AR‐LDH and EBT‐LDH. Thermodynamic parameters were calculated according to Equations (9) and (10).

(9) $$\Delta G=-RT\ln k_{d}$$

(10) $$k_{d}=55.5K_{L}$$

where, Δ*G* is the Gibbs free energy, *R* is the universal gas constant, *T* is adsorption temperature, *k*_d_ is the distribution coefficient and *K*_L_ is the Langmuir constant as defined in Equation (3) [52]. For AR, the capacity increased with temperature, going from 326.5 mg g^−1^ at 25°C to 356.9 mg g^−1^ at 55°C. This increase is consistent with an endothermic process in which elevated temperatures enhance ion mobility and access to interlayer sites. The spontaneity of the reaction is confirmed by the negative Δ*G* value of −36.22 kJ mol^−1^ at 25°C. In contrast, the EBT‐LDH system displayed a non‐monotonic dependence on temperature. While *q* increased from 1800 mg g^−1^ at 25°C to a maximum of 2793.2 mg g^−1^ at 35°C, it saw a subsequent decline to 2216.9 mg g^−1^ at 55°C. This trend reflects two competing temperature‐dependent behaviors. Aromatic dyes such as EBT are known to aggregate at lower temperatures at sufficiently high concentrations [53], which would make the dye molecule sterically less capable of adsorption. The initial rise in *q* from 25°C to 35°C follows the dissociation of EBT aggregates into monomers [54]. The subsequent decrease in q is consistent with the expected exothermic nature of the chelation process itself. The adsorption of EBT was also spontaneous, as can be seen from a Δ*G* value of −37.2 kJ mol^−1^ at 25°C. It should however be noted that the capacity at 55°C is higher than 25°C despite the exothermic process.

The reusability and regeneration potential of an adsorbent is a critical parameter in its development cycle. The reusability efficiency of the LDH for the AR and LDH is depicted in Figure 7c,d respectively. Over three regeneration cycles, AR‐LDH system showed % removal of 74.93%, with the adsorption capacity *q* decreasing from 150 mg g^−1^ to 112.4 mg g^−1^. On the other hand, the EBT‐LDH system showed a drastic reduction in % removal from 87.43% for the first cycle to 44.47% for the third. This reduction can be attributed to the distinct adsorption mechanisms for the two dyes. AR primarily attaches to the LDH via electrostatic and ion exchange modes, which are easily reversible under the regeneration conditions used here. EBT additionally undergoes a chelation bonding at the divalent metallic centers, and incomplete removal of these bonds is expected to leave a fraction of the chelated EBT on the adsorbent over subsequent cycles, which leads to a much‐lowered reusability. Other studies utilizing LDH as dye adsorbents have also reported similar reductions in efficiency over multiple cycles [26, 55].

### 3.3 DFT Calculations

**FIGURE 8:**
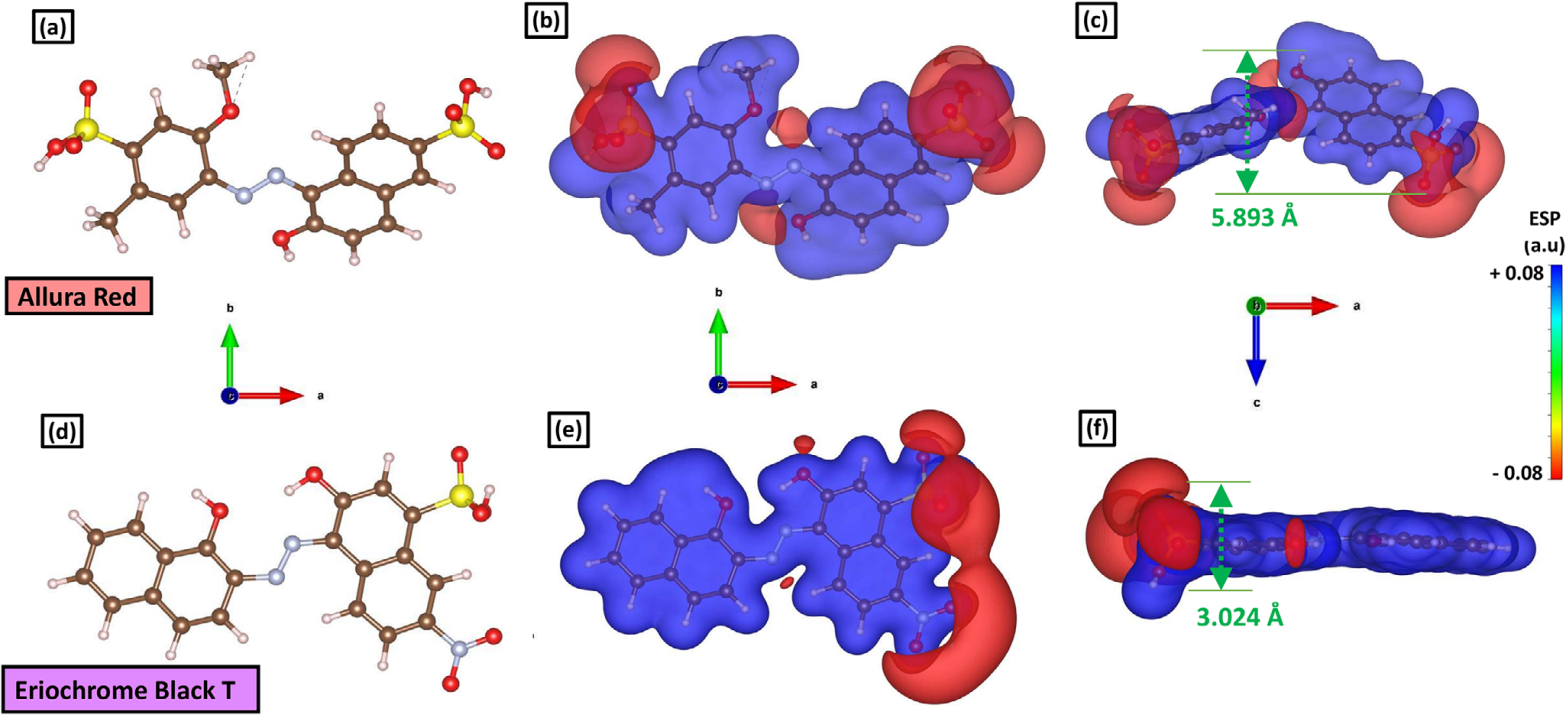
DFT‐optimized molecular structures and electrostatic potential (ESP) maps of azo dyes: (a–c) Allura Red (AR) and (d–f) Eriochrome Black T (EBT).

DFT calculations were performed to quantify the intrinsic charge distribution and geometric anisotropy of representative azo dyes. The ESP maps reveal pronounced differences in charge distribution between AR and EBT. For AR (Figure 8b,c), the negative electrostatic potential is broadly distributed across the molecular framework, spanning multiple functional groups and aromatic rings. This relatively uniform charge dispersion results in reduced charge anisotropy, indicating that electrostatic interactions with the LDH surface are likely to be more spatially delocalized. Consequently, AR exhibits a comparatively diffuse electrostatic interaction landscape, which may limit directional binding or site‐specific anchoring.

In contrast, EBT (Figure 8e,f) displays a highly localized negative electrostatic potential concentrated around the sulfonate and oxygen‐rich functional groups, while the conjugated aromatic backbone exhibits a relatively positive potential. This clear spatial separation of charge indicates strong electronic anisotropy. The side‐view ESP representation shown in Figure 8f further highlights this anisotropy, suggesting a strong propensity for directional electrostatic interactions with positively charged LDH layers. Such charge localization is expected to promote preferential adsorption orientations.

Beyond electronic effects, geometric anisotropy, quantified here by the effective molecular thickness, plays a decisive role in governing dye–LDH interactions. From the optimized DFT geometries, AR exhibits a substantially larger effective thickness of approximately 5.89 Å, whereas EBT adopts a more compact, planar conformation with a thickness of only ≈3.02 Å. This nearly twofold difference has important implications for adsorption and intercalation within the positively charged interlayers of LDH. The thinner, more planar EBT molecule can approach the LDH basal surface more closely, enabling stronger electrostatic coupling between the localized sulfonate groups and the positively charged metal hydroxide layers. Such geometric compatibility favors efficient interfacial contact and facilitates intercalation within the confined LDH galleries. In contrast, the bulkier AR molecule experiences steric constraints that limit its proximity to the LDH surface, resulting in partial charge screening and reduced effective interaction despite its distributed negative potential.

### 3.4 Adsorption Mechanisms

The exceptional performance of the NiCuAl LDH for the adsorption of AR and EBT dyes is the result of synergistic interactions of multiple physical and chemical mechanisms. Based on the material characterization and analysis of the isotherm and kinetic model fitting, we propose a multifaceted mechanism involving electrostatic attraction, ion exchange, hydrogen bonding, and metal chelation.

Electrostatic attraction serves as the primary driving force for the dye uptake in both systems. At the optimal pH of 3, the electrostatic attraction between the highly positive LDH layers and anionic functional groups of the dyes serves as the initial ‘long‐range’ force for pulling dye molecules from solution and onto the adsorbent surface [56]. The higher adsorption for EBT compared to AR can partially be explained by greater electrostatic forces in the case of the former due to the highly localized negative potential in EBT (Figure 8e). Beyond electrostatic forces, hydrogen bonding serves as a secondary mechanism. The abundant ─OH functional groups on the LDH surface (identified in the XPS survey spectrum in Figure 3) interact with the azo nitrogen atoms (─N═N─) as well as sulfonate (─SO_3_H) and hydroxyl (─OH) functional groups present in the dye molecules (Figure S1). For AR, the presence of the bulky methoxy group (─OCH_3_) introduces steric hindrance, which limits the effect of H‐bonding [57]. On the other hand, the presence of dual ─OH groups in EBT gives rise to a higher density of H‐bonding sites for the LDH. This disparity can be correlated to the differences in the initial linear region of the IPD plots as discussed in the previous section.

A high d‐spacing (8.58 Å) is a distinguishing feature of the synthesized LDH. This is the result of the intercalation of both nitrate (NO_3_^−^) and carbonate (CO_3_^2−^) ions in the interlayer spacing. To confirm this, we synthesized LDH by maintaining the metal nitrate amounts and increasing the amount of urea (17.5 mmol vs the originally used 6 mmol). The XRD patterns of both LDHs are presented in Figure S5. The (003) peak occurs at 2θ = 11.6° for the control LDH, which translates to a *d*‐spacing of 7.6 Å. This is due to the relative amounts of NO_3_^−^/ CO_3_^2−^ in the respective interlayers. In the presence of higher amounts of urea, the CO_3_^2−^ anions preferentially intercalate due to a stronger affinity with the LDH layers compared to NO_3_^−^ anions. In contrast, the lower urea amount leads to the presence of both NO_3_^−^ and CO_3_^2−^ anions in the interlayer. The structure of the LDH is left intact, as indicated by the non‐shifting of the (110) plane, which represents the cation–cation distance (dashed lines). NO_3_^−^ anions possess a lower charge density, which makes them easier to displace [58]. Moreover, the higher hydration size of NO_3_‐ leads to a larger interlayer opening, facilitating easier access to the adsorbing molecules. From the DFT calculations presented in the preceding section, the molecular thickness of AR is 5.89 Å compared to a thickness of 3.02 Å for the EBT. The larger LDH opening is thus particularly favorable for the EBT dye where it receives a clearance of 5.56 Å, as it can enter the interlayer longitudinally due to its lower effective thickness, as shown in Figure 8f. The high capacity observed here therefore reflects the substantial density of active sites distributed throughout the expanded interlayer volume, rather than multilayer stacking of dye molecules on top of one another.

**FIGURE 9:**
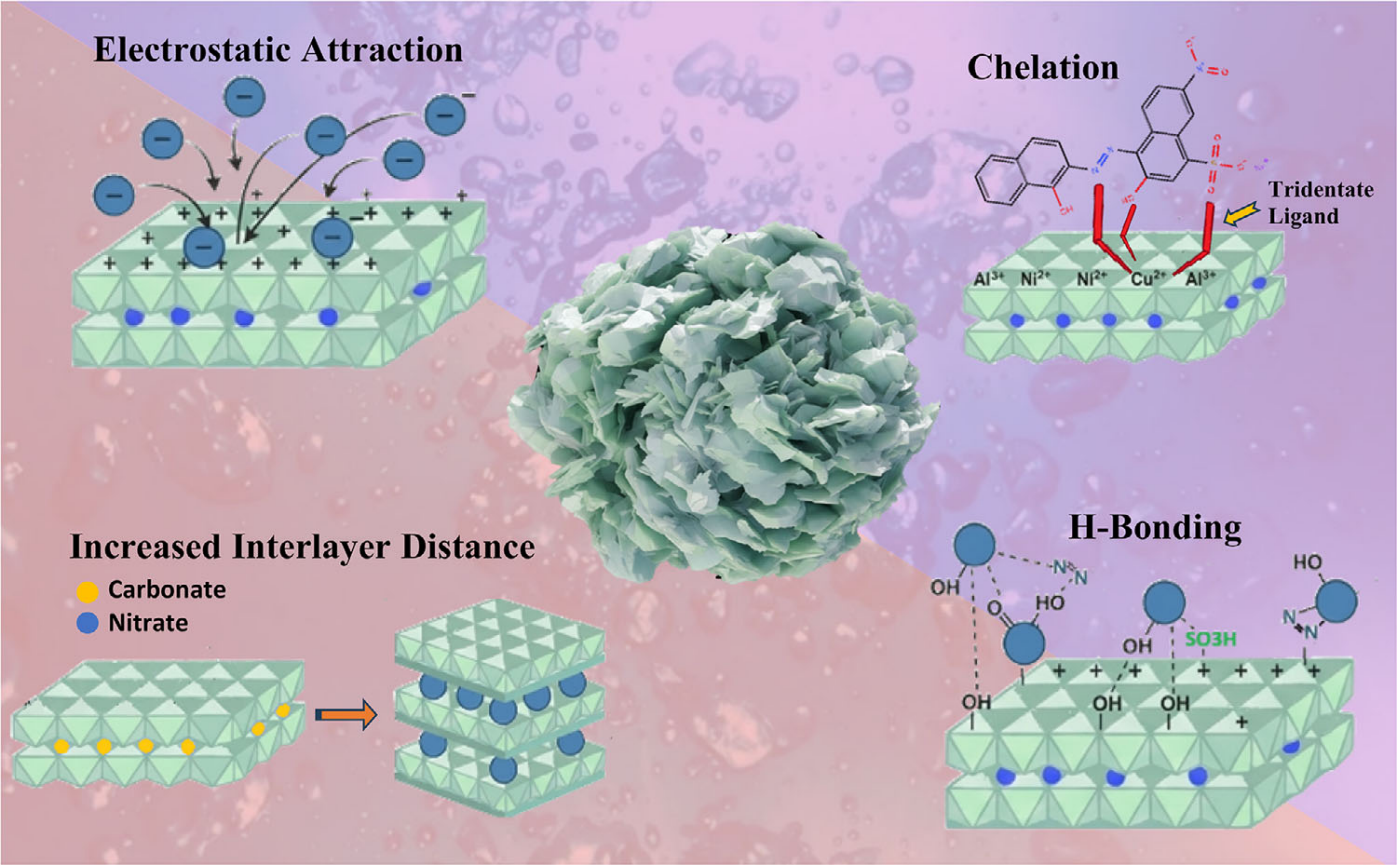
Possible dye adsorption mechanisms for Allura Red and Eriochrome Black T onto NiCuAl LDH.

The *q*_max_ for the two systems demonstrates that EBT‐LDH significantly outperforms the AR‐LDH system. In addition to the factors mentioned above, this disparity is primarily driven by the specific ability of EBT to undergo chelation. EBT forms a tridentate ligand by virtue of the hydroxyl group adjacent to the azo and sulfonic acid groups [6, 59]. This allows the EBT to form highly stable chelate rings with the transition metal like Ni^2+^ and Cu^2+^ [59]. Conversely, although AR also possesses a hydroxyl group and an azo linkage, the bulky ─OCH_3_ group hinders the formation of similar multidentate complexes. This chelation effect is the major driving force behind the higher efficiency of EBT adsorption onto LDH. The overall mechanism for the dye adsorption process is shown in Figure 9.

Table 3 summarizes the reported adsorption values for synthetic dyes for various LDHs. The CuNiAl LDH presented in this study material outperforms most of the materials. Therefore, our material can be considered a strong candidate for the remediation of wastewater polluted with various anionic dyes.

**TABLE 3:** Comparison with reported *q*_max_ values for various LDHs against synthetic dyes.

Material	Target dye	Isotherm model	Kinetic model	*q*_max_ (mg g^−1^)	Refs.
CuFe LDH	Eriochrome Black T	Freundlich	PSO	250	[20]
NiCoZn LDH	Rose Bengal	Langmuir	PSO	29.8	[60]
NiAlTi LDH	Methyl Orange Orange II	Langmuir	PSO	1250 2000	[15]
Graphene/NiMgAl LDH‐calcined	Eriochrome Black T	Langmuir	PSO	384.6	[16]
MgAl LDH	Acid Orange 7 Methylene blue	Langmuir Freundlich	Elovich	485.6 58.3	[13]
ZnAl/graphene oxide	Methyl orange	Langmuir	PSO	505.1	[61]
ZnMgAl LDH	Crystal violet	Langmuir	PSO	64.8	[45]
ZnAl LDH	Tartrazine	Langmuir	PSO	282.5	[26]
MgAl LDH	Methyl orange	Langmuir	—	846.6	[14]
MgCo LDH	Congo Red Acid Red 66	Langmuir	PSO	1050.3 285.4	[62]
Starch/MgAl LDH	Amaranth Eosin yellow Sunset yellow Tartrazine	Langmuir	PSO	167.2 150.9 154.7 162.9	[63]
MgAl LDH	Direct blue Reactive yellow Acid red Basic blue Disperse red	Langmuir Langmuir Freundlich Langmuir Langmuir	PSO	707.8 392.9 137.3 165.1 249.2	[64]
NiCuAl LDH	Allura red Eriochrome Black T	Langmuir	PSO	567 6628	This work

## 4 Conclusions

In this study, we successfully developed a ternary NiCuAl LDH that exhibits exceptional adsorption performance toward anionic pollutants. Our findings reveal that the hydrothermal synthesis yielded a mesoporous microstructure with a significantly expanded interlayer space (8.58 Å), which is paramount for overcoming the mass‐transfer limitations typically associated with bulky azo dyes. The profound disparity in adsorption capacity, where EBT (6628 mg g^−1^) vastly outperformed AR (567 mg g^−1^), highlights the importance of molecular‐level chemical affinity over simple surface area. We have discussed that while electrostatic attraction and hydrogen bonding serve as common mechanisms for both systems, the superior uptake of EBT is governed by a specific surface chelation mechanism. The ability of EBT to function as a tridentate ligand with the Ni^2+^ and Cu^2+^ cationic centers provides an advantage that AR lacks due to the steric hindrance of its methoxy group. Notably, thermodynamic data confirms spontaneous adsorption for both dyes at 25°C.

Furthermore, kinetic behavior differed markedly between the two dye systems. EBT, whose DFT‐derived molecular thickness (3.02 Å) leaves substantially greater interlayer clearance, reached equilibrium within 180 min, while AR, constrained by a larger molecular thickness of 5.89 Å and correspondingly limited clearance, exhibited slower, diffusion‐limited uptake with an equilibrium time of 800 min. Reusability testing over three adsorption‐desorption cycles showed different capacity retention between the two systems (75% capacity retained for AR versus a steeper decline for EBT), consistent with the comparative ease of reversing electrostatic binding relative to coordinate chelate bonds. While these results establish NiCuAl LDH as a highly efficient adsorbent under controlled laboratory conditions, practical deployment in real wastewater matrices would require further evaluation. Natural water systems typically contain co‐existing anions such as sulfates, phosphates, and chlorides, which are known to compete with target dyes for interlayer exchange sites and may reduce removal efficiency relative to the single‐dye systems studied here. Evaluation of adsorption performance in the presence of competing ions and natural organic matter represent important directions for translating this material toward field application. This work not only provides highly efficient material for environmental cleanup but also offers a mechanistic insight into the rational design of mixed‐metal adsorbents based on compositional and structural considerations.

## Author Contributions

Conceptualization: M.V. and H.L. Methodology: M.V., R.S., and H.L. Investigation and Formal Analysis: M.V., R.S., H.K., and Y.S.L. Visualization: M.V., H.K., and Y.S.L. Writing – Original draft: M.V., R.S., and H.K. Writing – review & editing: H.K., Y.S.L., and H.L. Resources: H.L.

## Conflicts of Interest

The authors declare no conflicts of interest.

## Supporting information




**Supporting File 1**: gch270149‐sup‐0001‐SuppMat.docx.

**Supporting File 2**: gch270149‐sup‐0002‐FigureS1‐S5.zip.

## Data Availability

The data that support the findings of this study are available from the corresponding author upon reasonable request.
