# Supplementary material for: Role of Interlayer Expansion and Surface Chelation in Ternary NiCuAl Layered Double Hydroxides for Ultrahigh Capacity Dye Adsorption
Source: Glob Chall. 2026 Aug 21;10(8):e70149. doi: 10.1002/gch2.70149 (PMC13495062; doi:10.1002/gch2.70149)
Supplement: Supplementary file 1 [file GCH2-10-e70149-s002.docx]

**Role of Interlayer Expansion and Surface Chelation in Ternary NiCuAl Layered Double Hydroxides for Ultrahigh Capacity Dye Adsorption**

**Figure S1:** Chemical structures of (a) Allura Red and (b) Eriochrome Black T

**Figure S2:** High magnification SEM image of NiCuAl showing platelet thickness

**Figure S3:** Pore size distribution from DFT method for NiCuAl LDH

**Figure S4:** pH_PZC_ curve for NiCuAl LDH

**Figure S5:** XRD patterns of NiCuAl LDH prepared with 6 mmol urea (red) and 17.5 mmol urea (green)
